# Toward Tailoring the Degradation Rate of Magnesium-Based Biomaterials for Various Medical Applications: Assessing Corrosion, Cytocompatibility and Immunological Effects

**DOI:** 10.3390/ijms22020971

**Published:** 2021-01-19

**Authors:** Philip Hartjen, Nils Wegner, Parimah Ahmadi, Levi Matthies, Ola Nada, Sandra Fuest, Ming Yan, Christian Knipfer, Martin Gosau, Frank Walther, Ralf Smeets

**Affiliations:** 1 Department of Oral and Maxillofacial Surgery, University Medical Center Hamburg-Eppendorf, Martinistr. 52, D-20246 Hamburg, Germany; l.matthies@uke.de (L.M.); m.yan@uke.de (M.Y.); c.knipfer@uke.de (C.K.); m.gosau@uke.de (M.G.); r.smeets@uke.de (R.S.); 2 Department of Materials Test Engineering (WPT), TU Dortmund University, Baroper Str. 303, D-44227 Dortmund, Germany; nils.wegner@tu-dortmund.de (N.W.); frank.walther@tu-dortmund.de (F.W.); 3 First Department of Medicine, Division of Infectious Diseases, University Medical Center Hamburg-Eppendorf, Martinistr. 52, D-20246 Hamburg, Germany; p.ahmadi@uke.de; 4 Department of Oral and Maxillofacial Surgery, Division of Regenerative Orofacial Medicine, University Hospital Hamburg-Eppendorf, D-20246 Hamburg, Germany; o.nada@uke.de (O.N.); s.fuest@uke.de (S.F.)

**Keywords:** magnesium, plasma electrolytic oxidation (PEO), microstructure, hydrogen evolution, cytocompatibility, immunological effects

## Abstract

Magnesium (Mg)-based biomaterials hold considerable promise for applications in regenerative medicine. However, the degradation of Mg needs to be reduced to control toxicity caused by its rapid natural corrosion. In the process of developing new Mg alloys with various surface modifications, an efficient assessment of the relevant properties is essential. In the present study, a WE43 Mg alloy with a plasma electrolytic oxidation (PEO)-generated surface was investigated. Surface microstructure, hydrogen gas evolution in immersion tests and cytocompatibility were assessed. In addition, a novel in vitro immunological test using primary human lymphocytes was introduced. On PEO-treated WE43, a larger number of pores and microcracks, as well as increased roughness, were observed compared to untreated WE43. Hydrogen gas evolution after two weeks was reduced by 40.7% through PEO treatment, indicating a significantly reduced corrosion rate. In contrast to untreated WE43, PEO-treated WE43 exhibited excellent cytocompatibility. After incubation for three days, untreated WE43 killed over 90% of lymphocytes while more than 80% of the cells were still vital after incubation with the PEO-treated WE43. PEO-treated WE43 slightly stimulated the activation, proliferation and toxin (perforin and granzyme B) expression of CD8^+^ T cells. This study demonstrates that the combined assessment of corrosion, cytocompatibility and immunological effects on primary human lymphocytes provide a comprehensive and effective procedure for characterizing Mg variants with tailorable degradation and other features. PEO-treated WE43 is a promising candidate for further development as a degradable biomaterial.

## 1. Introduction

Magnesium (Mg) is an abundant lightweight metal that has been extensively studied as a biomaterial for degradable implant applications in recent years [1,2]. Mg is a natural and essential component of the human body in its ionic form Mg^2+^. Moreover, its mechanical properties resemble those of human cortical bone and its compressive yield strength and elastic modulus are closer to those of natural bone than is the case for other commonly used metallic biomaterials [1]. Therefore, magnesium-based biomaterials offer promising prospects for applications in regenerative medicine that benefit from short- to medium-term stability and long-term degradability. However, unmodified magnesium is not well suited for most biomedical areas of application due to its rapid degradation in aqueous milieus with concomitant alkalization and hydrogen gas evolution in tissues surrounding the site of implantation, as well as loss of stability [3,4,5,6,7,8,9]. Possible solutions to slow down the degradation rate of magnesium include various alloying and surface modification approaches [2,10]. One surface modification technique that has been shown to decelerate magnesium degradation while simultaneously yielding biocompatible and microstructured surfaces is plasma electrolytic oxidation (PEO), also known as micro arc oxidation (MAO) [11,12]. PEO is an electrochemical process that causes Mg to react with the substrate electrolytes, which results in the formation of a surface ceramic layer. By varying the electrolyte composition and/or electrochemical process parameters, surfaces with various physical and chemical characteristics can be generated [13,14,15]. Notably, as the degradation rate depends on the composition and depth of the PEO-layer, the degradation kinetics could be tailored, within limits, towards specific purposes [11]. Moreover, the surface-topography of the microstructures of PEO-generated surfaces is affected by the electrochemical parameters and electrolytes used in the PEO-process [11,16]. Thus, the microstructural characteristics could be designed to optimize attachment of target tissue cells.

Mg variants that are developed as biomaterial candidates need to undergo comprehensive characterization regarding their chemical, mechanical and biological properties. Measuring the corrosion rate and the cytocompatibility is of key importance in this regard. Here, a PEO-treated Mg WE43-based biomaterial candidate is characterized by microstructural analysis of the surface, determination of hydrogen evolution in immersion tests, assessment of cytocompatibility and an assessment of effects in immune cells using primary human lymphocytes.

## 2. Results

### 2.1. Macro- and Microstructure

#### 2.1.1. Mg WE43 MEO

**Figure 1:**
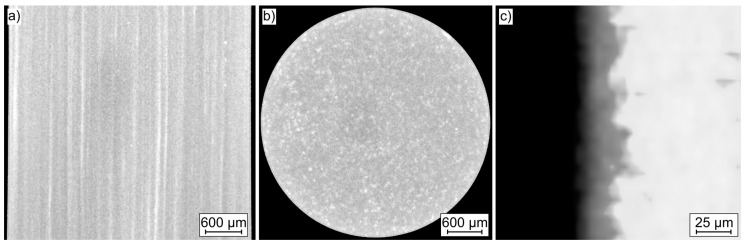
µ-Computed tomography images of cylindrical specimens: (**a**) longitudinal section of Mg WE43 MEO specimen, (**b**) cross section of Mg WE43 MEO specimen, (**c**) longitudinal section of Mg WE43 MEO plasma electrolytic oxidation (PEO) layer.

Figure 1 shows the reconstructed µ-computed tomography (µCT) volume of a Mg WE43 MEO cylinder in the cross and longitudinal section, as well as the longitudinal section, of the PEO coating. With the achieved resolution, no defects in the form of pores in the substrate material (a) and (b) can be detected. Due to the gray value differences, pores larger than 6 µm would be visible. In both images, a distinctive macrostructure is recognizable. In the cross section (b), brighter areas are discernible over the entire surface, which has a proximate round shape. Due to the contrast differences, these areas can be assigned to alloying elements with a higher density than those of the magnesium matrix. No differences are visible between the core and the edge area of the cylinder. In the longitudinal section (a), the specimen exhibits a different macrostructure. In contrast to the cross section, the brighter areas are distributed as lines parallel to the extrusion direction, so that an enrichment of the alloying elements is assumed. Consequently, the distribution of the alloying elements is attributed to the manufacturing process [17]. Analogous to the cross section, no differences between the core and edge areas can be observed. Panel (c) shows the characteristic interface between the PEO coating and the substrate material. Due to the uneven interface, the layer thickness varies between 15 and 30 µm. Further statements about the layer morphology regarding pores and cracks cannot be made based on these images.

**Figure 2:**
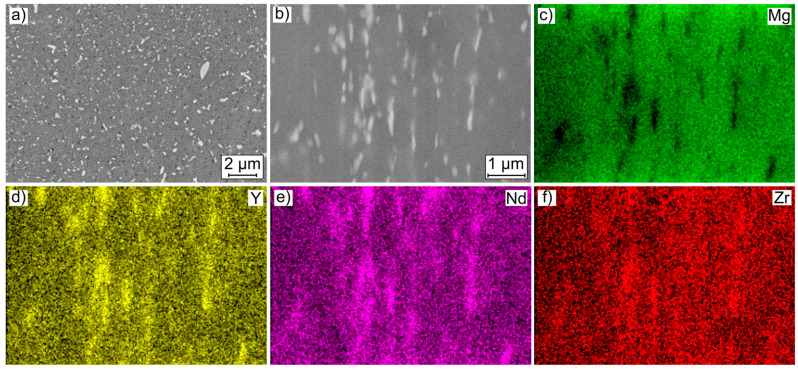
Results of microstructural investigations on Mg WE43 MEO: (**a**) SEM images of cross section; (**b**) SEM images of longitudinal section for EDS mapping of (**c**) Mg, (**d**) Y, (**e**) Nd and (**f**) Zr.

Figure 2 shows a cross sectional and longitudinal microstructural image and the corresponding energy dispersive X-ray spectroscopy (EDS) mappings of magnesium (Mg), yttrium (Y), neodymium (Nd) and zirconium (Zr).

Both microstructural images confirm the observations of the µCT scans. Finely distributed precipitates are discernible over the entire area, with the orientation of the precipitates varying between the cross and the longitudinal sections. The cross section reveals precipitates of different sizes, shapes and orientations. The size varies between a few 100 nm and about 1 µm. The larger precipitates have a stick shape with no preferred orientation. In the longitudinal section, the precipitates have a similar size, but they are arranged in lines. Analogous to the µCT images, the alignment of the precipitates corresponds to the extrusion direction. It is not possible to differentiate the precipitates based on their contrast. For further characterization of the microstructure, the EDS mappings of the scanning electron microscope (SEM) image are considered. A focus is set on the longitudinal section since this describes the condition on the lateral surface of the specimens. The brighter areas in the SEM image are visible in the Mg mapping as darker and thus as magnesium poor areas. At these sites, accumulations of yttrium and neodymium can be observed in the corresponding element mappings, indicating intermetallic precipitates. It can therefore be assumed that these are not pure yttrium or neodymium precipitates, but yttrium-rich and/or neodymium-rich precipitates. This also explains why, despite the different atomic numbers of the two elements, the contrast barely differs. For zirconium, only single punctual sites with an increased occurrence of the element are visible. Based on the EDS mappings, only qualitative statements can be made. A quantitative analysis of the precipitates was not carried out, as no reliable statement can be made due to the small size of the precipitates and the range of the X-ray generation. The size of the range depends on the respective elements to be examined as well as the energy of the electron beam, and an influence of the matrix material on the analysis results cannot be excluded [18]. Consideration of other studies allows a quantitative estimation of the phases present. Besides the matrix (α-Mg), it consists of a second phase Mg_12_(RE), located at the grain boundaries, a stick-like β-phase within the matrix (Mg_14_Nd_2_Y), and yttrium-rich (MgY, Mg_14_Y_5_, Mg_24_Y_5_), neodymium-rich (Mg_41_Nd_5_, Mg_12_Nd), and zirconium-rich precipitates [19,20,21].

#### 2.1.2. Plasma Electrolytic Oxidation Coating

**Figure 3:**
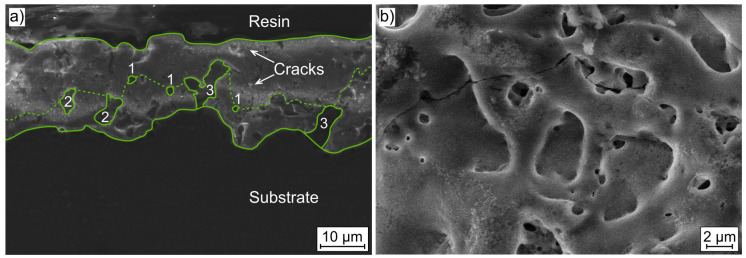
Results of microstructural investigations of plasma electrolytic oxidation (PEO) coating on Mg WE43 MEO: (**a**) SEM image of cross section: (1) circular pores, (2) arbitrarily formed pores, (3) arbitrarily formed pores with connection to the substrate material (**b**) SEM image of top view.

Figure 3 displays SEM images of the longitudinal section and a top view of the PEO coating. The sectional view shows the characteristic high porosity of this coating type. Pores of different shapes and sizes, as well as cracks, are visible. The pore size varies between 1 and 10 µm. The figure shows a classification depending on the shape, size and location of the pores. Type 1 describes circular pores, which usually have a diameter of 1 to 2 µm. In contrast, types 2 and 3 have an arbitrary, branched shape, whereas type 3 has a direct connection to the substrate material. Furthermore, the majority of the pores occur at a distance of approximately 10 µm from the substrate (dotted line). The interface between the coating and the substrate material shows an uneven texture so that the coating thickness also varies. The top view shows further, smaller pores besides the already mentioned pore sizes as well as the high surface roughness associated with this manufacturing process.

#### 2.1.3. Corrosion Behavior

**Figure 4:**
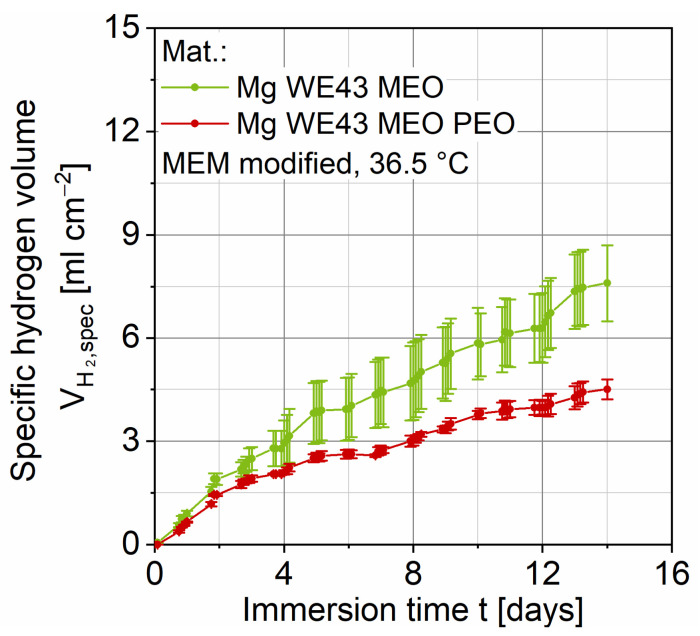
Results of immersion tests on Mg WE43 MEO and WE43 MEO PEO in a modified minimum essential medium at 36.5 °C and an immersion time of up to 14 days.

Figure 4 presents the summarized results of the immersion tests with the specific hydrogen volume $V_{H_{2},\mathrm{spec}}$ plotted over the immersion time t. The individual data points as well as the respective standard deviations are shown. Three tests are averaged for each of the two test series. In the beginning, the hydrogen evolution of Mg WE43 MEO increases linearly, after two days the slope decreases until it seems to increase after about twelve days. The shown standard deviations are on a relatively high level and increase with increasing immersion time. In comparison, the absolute values of the PEO coating are significantly lower and the qualitative course also differs. After an almost linear increase at the beginning, the slope of the curve decreases constantly. The standard deviations are on a significantly lower level than those of the Mg WE43 MEO tests, but also increase with increasing immersion time. The specific hydrogen volume (mean value) after two weeks for the PEO coating is 40.7% below the Mg WE43 MEO, which is in accordance with the manufacturer’s specification of at least 30% reduction within the first twelve weeks.

#### 2.1.4. Cytocompatibility

**Figure 5:**
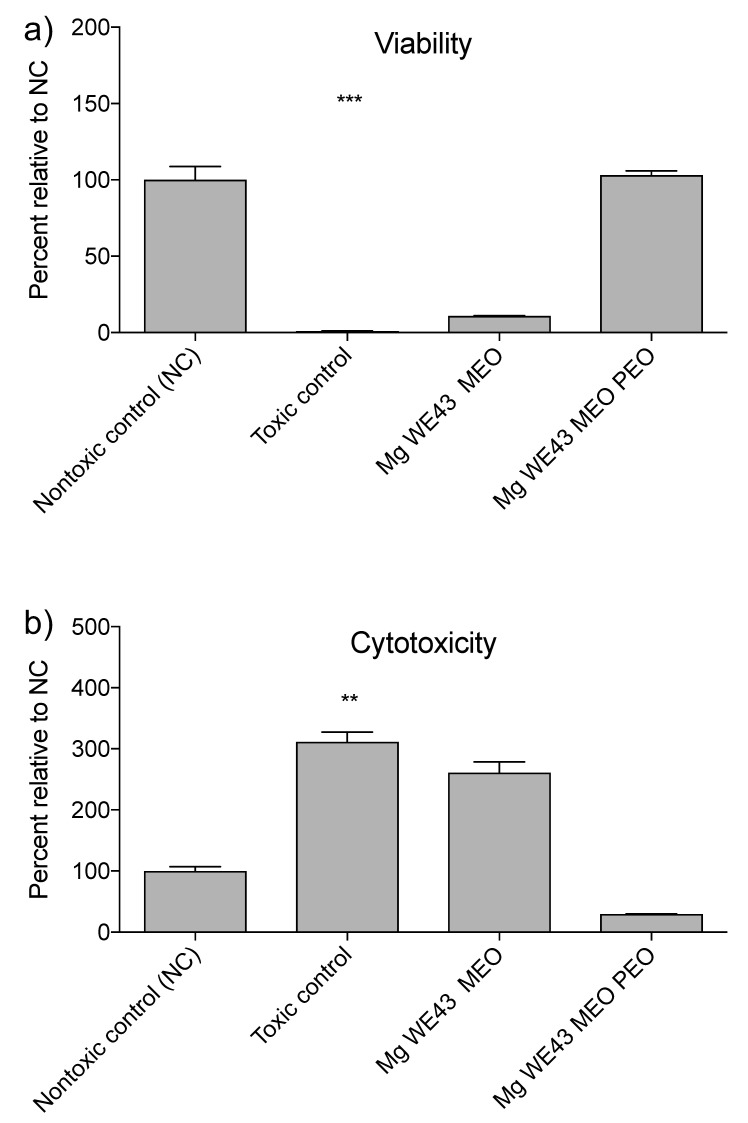
Extract assay results, (**a**) viability, (**b**) cytotoxicity. Asterisks show the results of Kruskal–Wallis tests followed by Dunn’s multiple comparison tests and indicate values that are significantly different from the nontoxic control. **: *p* ≤ 0.01 and ***: *p* ≤ 0.001.

Compared to the nontoxic control, cells cultured in the extract of Mg WE43 MEO show reduced viability (11% of the nontoxic control), while cells cultured in the Mg WE43 MEO PEO extract show no decreased viability (Figure 5a). Mg WE43 MEO exhibits cytotoxicity, analogous to the toxic control (around 300%) (Figure 5b), while Mg WE43 MEO PEO shows no cytotoxicity.

**Figure 6:**
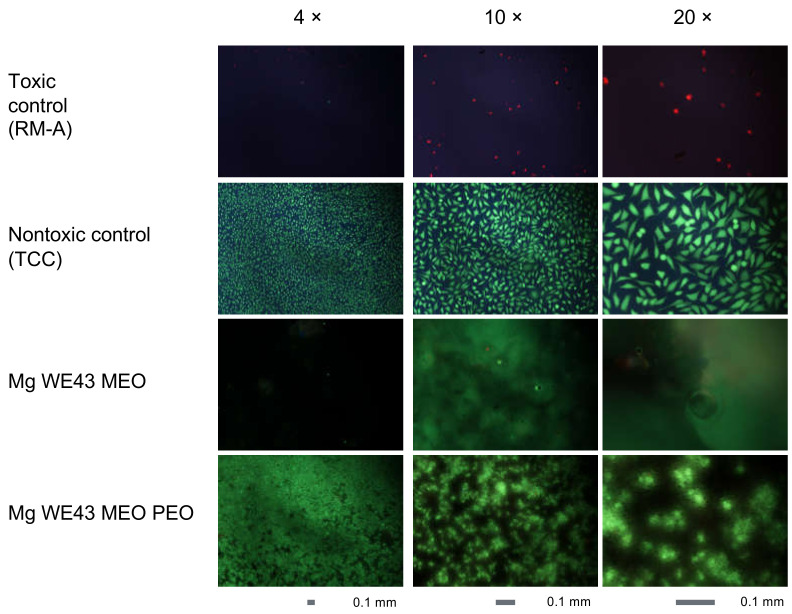
Live/dead staining assay results for L-929 cells that were directly seeded and cultured for 24 h on the material specimens. Nuclei of dead cells with compromised plasma membrane integrity are stained red by propidium iodide while the green dye fluorescein diacetate exclusively stains viable cells. The scale bars indicate a length of 0.1 mm.

In line with the results of the extract assays, cells on Mg WE43 MEO show a rounded morphology and are strongly reduced in number compared to the nontoxic control material (Figure 6). On the other hand, cells visualized on Mg WE43 MEO PEO show a less rounded morphology and no obvious reduction in number, although their morphology is less elongated and spindle-shaped than that of cells cultured on the negative control material. The images of Mg WE43 MEO PEO appear blurred and granular in comparison to the images of the negative control material.

#### 2.1.5. Effects on Primary Human Lymphocytes

**Figure 7:**
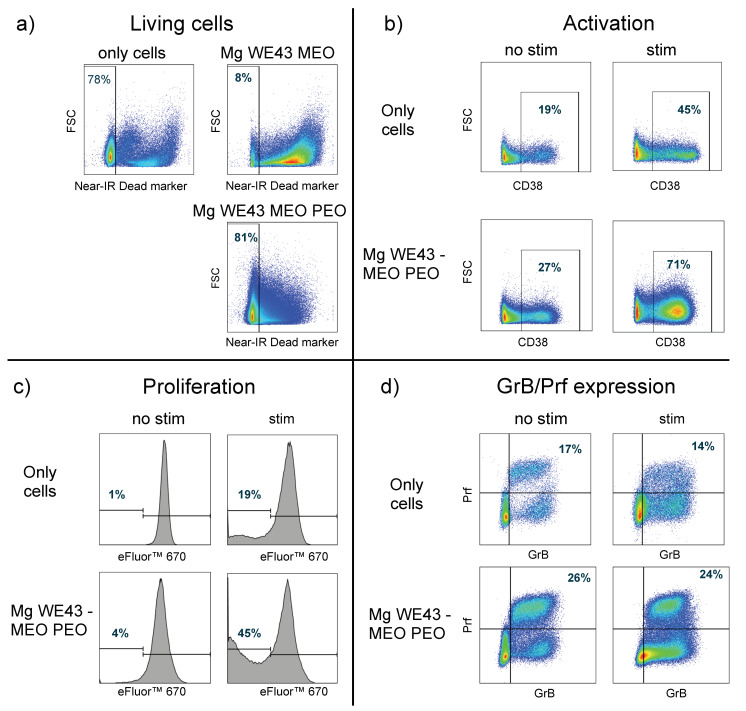
Effects on primary human lymphocytes: (**a**) living cells among total PBMC, detected using a fixable near-IR viability kit (Thermo Fischer, Waltham, MA, USA) (**b**) activation of CD8^+^ T cells (measured by determining the surface expression of CD38), (**c**) proliferation of CD8^+^ T cells (measured by determining the number of cells with low fluorescence intensity for the proliferation tracking dye eFluor™ 670. The dye is distributed equally between daughter cells upon cell division, thus, proliferation can be measured as successive reduction of the fluorescence intensity of the cells), (**d**) expression of granzyme B and perforin on CD8^+^ T cells. (**b**–**d**) show representative plots of two experiments with at least 200,000 cells acquired per setting in each experiment.

As expected, cells incubated in the presence of untreated Mg WE43 MEO show a drastic reduction in the number of living cells (only 8% are alive), while cells incubated with PEO-treated Mg WE43 MEO show a similar number of living cells (81%) as cells alone (78%) (Figure 7a). Therefore, cells incubated with untreated Mg were not analyzed further. CD8^+^ T cells within the PBMC incubated with PEO-treated Mg are more activated. This is the case for unstimulated cells (27% of the cells incubated with Mg PEO are CD38^+^, compared to 19% for cells alone) and for stimulated cells (71% vs. 45%) (Figure 7b). Moreover, the cells that were incubated with PEO-treated Mg showed increased proliferation with 4% vs. 1% and 45% vs. 19% for unstimulated and stimulated cells, respectively (Figure 7c). Furthermore, PBMC that were incubated with PEO-treated Mg showed higher proportions of GrB^+^ and Prf^+^ CD8^+^ T cells—26% vs. 17% of the cells were double positive in the case of unstimulated cells and 24% vs. 14% in the case of stimulated cells (Figure 7d).

## 3. Discussion

This study aimed to investigate a PEO-surface on the magnesium alloy WE43 MEO as a candidate for biomaterial applications in vitro, regarding surface characteristics and hydrogen gas evolution, as well as cytocompatibility.

The microstructure of both materials under consideration differs significantly and is influenced by the respective manufacturing process. While for Mg WE43 MEO the structure is dominated by a high number of finely distributed precipitates of the alloying elements, the PEO layer shows a high number of pores and microcracks, as well as a high surface roughness. For Mg WE43 MEO, intermetallic precipitates are formed which are deformed along the process direction as a result of extrusion [17]. Rare earth elements are considered to have a passivating effect on the corrosion behavior. This effect is intensified when the elements are finely distributed over the entire surface [22]. In contrast, the PEO layer itself leads to an increase in corrosion and wear resistance [23]. The production process is controlled by the generation of a plasma environment due to a high applied voltage and the subsequent plasma thermochemical interactions, including dielectric discharge [24]. The present layer shows the typical rough-wave interface, which leads to a good integration of the layer into the substrate material [25,26]. In particular, the pores with connection to the substrate material (type 3) are formed as a result of the mentioned dielectric discharge [27], but like the microcracks, they do not penetrate the entire layer. A high surface roughness results from the manufacturing process and the pores.

For a first comparison of the PEO coating with the untreated substrate material, the results of the immersion tests are discussed. The course of the magnesium alloy shows a decrease of the slope and thus of the hydrogen evolution rate with increasing immersion time so that passivation due to the rare earth elements is suspected [22]. This is accompanied by an enrichment of the oxide layer by these elements resulting in a changed Pilling–Bedworth ratio (PBR) [28]. This is defined as the ratio of the molar volume of the metallic element and its oxide and is 0.81 for pure magnesium. PBR values < 1 lead to an unstable oxide layer and therefore cannot provide sufficient protection. As a result of alloying elements, this value can be slightly changed, and the passivation behavior can be affected [29]. The qualitative progression is comparable with other studies on the corrosion behavior of Mg WE43. Analogous to these studies, the course can be divided into three sections. In the beginning, the hydrogen volume increases with an almost constant hydrogen evolution rate (HER) and, subsequently, the slope decreases steadily. This is accompanied by the formation of a corrosion layer, which has a passivating effect and thus leads to a reduction in hydrogen evolution. At the end of the measurement, the HER seems to increase again. According to current studies, this is due to the formation of local corrosion pits and a disruption of the corrosion layer at these spots [30,31]. In comparison, Mg WE43 MEO PEO shows qualitatively and quantitatively deviating corrosion behavior. After an initial phase with an approximately constant hydrogen evolution rate, the slope of the curve decreases continuously until an approximately constant HER is reached. The absolute values are significantly below those of the substrate material. In general, PEO coatings increase corrosion resistance and thus reduce hydrogen evolution [24,32]. Based on the results of Jung et al., a phosphate-based electrolyte was used for the coating process in this study [33]. In particular, phosphate-based coatings lead to a compact inner layer area; the structure images (Figure 3) show just this compact inner layer. According to Duan, the nature of this inner layer determines the characteristic corrosion behavior, and compact layers are accompanied by an increase in corrosion resistance [25].

PEO treatment led to a clear improvement of the cytocompatibility of Mg WE43 MEO. Viability and cytotoxicity are similar for Mg WE43 MEO PEO and the negative control in the extract assays, while untreated Mg WE43 MEO is clearly cytotoxic. In the live/dead staining assay, very few cells are visible on untreated Mg WE43 MEO because dead cells do not firmly attach to the surface and are rinsed off during the staining and washing steps of the staining procedure. In contrast to untreated Mg WE43 MEO, no obvious cytotoxicity was detected for the PEO-treated variant in the direct live dead staining assay. The blurred and granular appearance of the fluorescence microscopy images of Mg WE43 MEO PEO in the live/dead staining assay likely results from the high porosity of its surface (Figure 3). After incubation for three days, untreated WE43 killed over 90% of lymphocytes, while more than 80% of the cells were still vital after incubation with the PEO-treated WE43. PEO-treated WE43 MEO slightly stimulated the activation, proliferation and toxin (perforin and granzyme B) expression of CD8^+^ T cells. This suggests that PEO-treated WE43 MEO may cause adverse immunological responses, and, therefore, we plan to characterize these effects in more detail in future studies in which we analyze additional subsets of immune cells such as monocytes, B-cell, NK-cells etc., as well as other (PEO)-surfaces. In a next step, it could be a promising approach to optimize Mg-based biomaterials specifically in a direction of favorable immunocompatibility using our flow cytometry-based assay.

Taken together, the results of the cytocompatibility assessment confirm that PEO treatment can greatly improve the cytocompatibility of Mg-based materials. The performed investigations show significant improvements for the PEO-treated WE43 MEO compared to the untreated substrate material. The corrosion rate was strongly reduced and the cytocompatibility was greatly improved.

## 4. Materials and Methods

### 4.1. Preparation of Material Specimens

**Figure 8:**
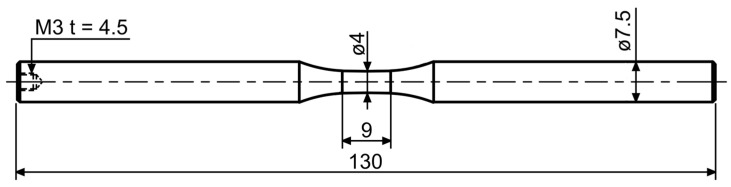
Specimen geometry for µ-computer tomography and immersion tests.

The raw material used was extruded magnesium of WE43 MEO alloy (yttrium 3.62–3.78%, neodymium 2.74–2.91%, zirconium 0.24–0.28%; Meotec, Aachen, Germany) with an initial diameter of 9.5 mm. Additionally, magnesium specimens were coated by plasma electrolytic oxidation (PEO, Kermasorb^®^, Meotec, Aachen, Germany). Based on the results of Jung et al., a phosphate-based electrolyte was used for the coating process [33]. For microstructural investigations, samples were separated from the raw material and embedded using a conductively cold embedding resin. These samples were then polished to 1 µm with water-free diamond suspension. For the immersion tests, cylindrical specimens according to Figure 8 were used. The area to be examined has an initial gauge length of 9 mm and an initial diameter of 4 mm. In this area, all specimens were polished to 1 µm. For the PEO-coated specimens, this treatment step was performed before the coating was applied. To ensure that only the measuring area corrodes, the rest of the specimen was coated with an anti-corrosion lacquer.

Cylindrical specimens were utilized for the cytocompatibility assessment. The Mg WE43 MEO specimens had a diameter of 4 mm and a height of 1 mm. The Mg WE43 MEO PEO specimens had a diameter of 6 mm and a height of 10 mm with a screw thread on the back side for contacting the electrode during PEO treatment. The screw threads are covered with plastic screws before subjecting the specimens to the cytocompatibility assessment.

### 4.2. Assessment of Macro- and Microstructure

For the evaluation of the macro-as well as the defect structure, scans were performed on cylindrical specimens (Figure 8) using a X TH 160 µ-computed tomograph (µCT, Nikon, Tokyo, Japan) with a maximum acceleration voltage of 160 kV. A beam energy of 105 kV, a beam current of 55 µA, a power of 5.8 W and an exposure time of 354 ms, as well as an effective pixel size of 6 µm, were used. The evaluation of the reconstructed volume was carried out by the software VGStudio Max 2.2 (Volume Graphics, Heidelberg, Germany). To characterize the microstructure concerning the phase distribution and precipitation condition, investigations were carried out using a scanning electron microscope (SEM, Crossbeam XB 550L, Zeiss, Oberkochen, Germany) equipped with a charge compensator. The elemental composition was analyzed by the integrated energy dispersive X-ray spectroscopy (EDS) unit.

The microstructure of the PEO coating was examined with a SEM (Mira 3 XMU, Tescan, Dortmund, Germany). A longitudinal section of the samples was investigated; the surface was treated analogously to the magnesium samples. The surface condition (top view) of the coating was examined on the cylindrical samples. An acceleration voltage of 10 kV and a working distance between 20 and 25 mm were used for the investigations.

### 4.3. Immersion Test

Immersion tests with an immersion time t of two weeks were carried out to assess the corrosion behavior of the substrate material Mg WE43 MEO as well as the PEO coating. As corrosion medium, a minimum essential medium (MEM, Life Technologies, Carlsbad, CA, USA) with the addition of 1% glutamine, as well as 1% penicillin/streptomycin, and 1% fungizone at 36.5 °C was used. To prevent contamination and saturation effects, the entire medium was changed every 48 h. At each exchange, the specimens were cleaned with distilled water and the corrosion cell with ethanol. For this purpose, specimens according to Figure 8 were utilized. During the corrosion process, the hydrogen gas produced was detected via a burette with a collecting funnel. The gas volume was related to the lateral surface in the measuring area.

### 4.4. Cell Culture

L-929 mouse fibroblasts (LGC Standards, Wesel, Germany) were cultured in minimum essential medium supplemented with 10% fetal bovine serum, penicillin/streptomycin (100 U/mL each) (all from Life Technologies, Carlsbad, CA, USA) and L glutamine (Sigma–Aldrich, St. Louis, MO, USA) to a final concentration of 4 mM at 37 °C, 5% CO_2_ and 95% humidity.

### 4.5. Reference Materials for the Cytocompatibility Assessment

As a toxic control, RM-A (Hatano Research Institute, Food and Drug Safety Center, Hadano, Japan) was used. For the live/dead staining assay, TC coverslips (Sarstedt, Nuembrecht, Germany) were used as a nontoxic control.

### 4.6. Cytocompatibility Assessment

The in vitro cytocompatibility assessment was carried out as described before [11] using indirect viability and cytotoxicity assays in combination with a direct live/dead staining assay. In brief, for indirect assays, two specimens of each type, as well as toxic control samples, were extracted under cell culture conditions (37 °C, 5% CO_2_ and 95% humidity) with cell culture medium at a ratio of 3 cm^2^/mL while cell culture medium incubated under identical conditions served as the negative control extract. The extracts were centrifuged at maximum speed in a benchtop centrifuge for 10 min and the supernatants were used as medium to culture cells for 24 h. Subsequently, cytotoxicity and viability were measured using LDH (BioVision, Milpitas, CA, USA) and XTT (Cell Proliferation Kit II, Roche Diagnostics, Mannheim, Germany) assay kits, respectively, according to the manufacturer’s manuals. The live/dead staining assay was carried out at a surface-to-volume ratio of 5.65 cm^2^/mL and the stained samples were visualized using an upright fluorescence microscope (Eclipse Ti–S/L100, Nikon, Duesseldorf, Germany), equipped with a filter for parallel detection of red and green fluorescence.

### 4.7. Assessment of Effects on Primary Human Lymphocytes

Peripheral blood samples were collected into BD Vacutainer^®^ CPT (TM) (BD Bioscience, Franklin Lakes, NJ, USA) containing 0.1 M sodium citrate and peripheral blood mononuclear cells (PBMCs) were isolated following the manufacturer’s instruction. PBMCs were then labeled with eFluor™ 670 (Thermo Fischer, Waltham, MA, USA) for proliferation tracking. Subsequently, unstimulated PBMCs as well as PBMCs stimulated with a CD3 antibody (purified OKT3) were cultured for three days in the presence or absence of biomaterial specimens at 37 °C and 5% CO_2_ at surface-area-to-volume ratios that ensured that the specimens were well covered with medium, about 1 cm^2^/mL for Mg WE43 MEO and 2 cm^2^ for Mg WE43 MEO PEO.

Staining with antibodies and flow cytometry was conducted as described elsewhere [34]. Briefly, PBMCs were washed and stained with a live/dead (TM) fixable near-IR viability kit (Thermo Fischer, Waltham, MA, USA) to identify live cells, as well as the following monoclonal surface antibodies at room temperature for 20 min: BV650 anti-CD8, Alexa Fluor^®^ 700 anti-CD3. Following washing, the PBMCs were fixed and permeabilized using Foxp3/Transcription Factor Staining Buffer Set (eBiosciencesTM, San Diego, CA, USA) for 45 min at 4 °C. After another washing step, the following intracellular antibodies were added and the cells were incubated for 30 min at 4 °C: BV421 anti-perforin, FITC anti-CD4, and PE/DazzleTM anti-granzyme B. All antibodies were purchased from Biolegend^®^ (San Diego, CA, USA) unless otherwise stated. Finally, the cells were washed and analyzed on a BD LSRFortessa (TM) (BD Bioscience). Flow cytometry data were analyzed using the software FlowJo 10 (Ashland, OR, USA). CD8^+^ T cells were gated as singlet, live, CD3^+^, CD4^™^, CD8^+^ lymphocytes.

### 4.8. Statistical Analysis

The indirect viability and cytotoxicity assay data were analyzed for statistically significant differences in comparison with the nontoxic control. For this purpose, Kruskal–Wallis tests followed by Dunn’s multiple comparison tests were performed using the software Graphpad Prism 7.0e (GraphPad software, San Diego, CA, USA).

## 5. Conclusions

In conclusion, this study demonstrates that the combined assessment of corrosion, cytocompatibility and immunological effects on primary human lymphocytes provides a comprehensive and effective procedure for characterizing Mg variants with tailorable degradation and other features. PEO-treated WE43 is a promising candidate for further development as a degradable biomaterial.

## Abbreviations

EDS	Energy dispersive X-ray spectroscopy
GrB	Granzyme B
HER	Hydrogen evolution rate
MEM	Minimum essential medium
PBR	Pilling-Bedworth ratio
PEO	Plasma electrolytic oxidation
Prf	Perforin
SEM	Scanning electron microscope
